# Frequency-Dependent Reaction of the Triceps Surae Muscle of the Mouse During Electromyostimulation

**DOI:** 10.3389/fphys.2020.00150

**Published:** 2020-02-28

**Authors:** Sebastian Zart, Joshua Berger, Oliver Ludwig, Janosch Knauth, Michael Fröhlich

**Affiliations:** Department of Sport Science, Technische Universität Kaiserslautern, Kaiserslautern, Germany

**Keywords:** electrostimulation, mice, muscle activation, whole-body EMS, frequency

## Abstract

The difference in the efficacy of altered stimulation parameters in whole-body-electromyostimulation training (WB-EMS) has hardly been examined. Higher impulse frequencies (>50 Hz) might be most adequate for strength gains because of the force frequency relationship (FFR), which describes a greater force production by increasing the applied frequency. Frequencies below this value, however, also seem to have positive influences on muscle strength increases. Therefore, the aim of this study was to analyze possible muscle length changes to different stimulation frequencies of the dissected mouse triceps surae muscle. A bending rod transducer was used to measure and compare changes in muscle lengths at different frequencies in relation to the initial length in the prepared muscle. We found significant differences between the muscle shortening at different frequencies (*p* < 0.001). At 20 Hz the largest muscle shortening was observed (20 Hz = 3.32 ± 2.06, 60 Hz = 0.77 ± 0.58, 85 Hz = 0.32 ± 0.29, 100 Hz = 0.31 ± 0.29). From a frequency of 60 Hz, the muscle shortening decreased progressively, at stimulation frequencies above 60 Hz the lowest shortenings were recorded. The results demonstrate a different behavior of the isolated triceps surae muscle of the mouse in an *ex vivo* environment. Even if there is no FFR in this investigation, the results indicate a higher metabolic demand using higher frequencies in electromyostimulation, despite the experimental execution in *ex vivo* design. Therefore, future studies should take this faster fatigue into account when drawing up training protocols in order to counteract possible frequency modulations.

## Introduction

Electromyostimulation (EMS) training has been an effective form of strength training for many years now, both in rehabilitation and in competitive and popular sports. However, there has been no clear consensus on the selection of stimulation parameters in training planning and execution over the last decades. A stimulation parameter whose influence on the effectiveness of EMS training has not yet been sufficiently clarified is the stimulation frequency. In conventional EMS training, a frequency in the range around 85 Hz is usually used, but there is no evidence for this. Various studies differentiate the frequency range used according to age or state of fatigue. Optimal force increases seem to take place at a frequency in the range of 76.4 ± 20.9 Hz (Filipovic et al., 2011). In general, more than 50 Hz seem to be necessary to generate optimal force increases, since at a frequency below 50 Hz mainly slower Type I fibers and from 50-120 Hz mainly faster Type II fibers are supposed to be stimulated (Frenkel et al., 2004). According to Kramme (2007), optimal faradic stimulation of the striated musculature occurs from 50 Hz, while Moreno-Aranda and Seireg (1981) were able to generate maximum electrical muscle activity at a frequency in the range between 50 and 110 Hz. However, frequency ranges below 50 Hz are also considered to have a positive influence on force increases. Dreibati et al. (2010) recommend a stimulation frequency below 60 Hz in order to prevent a loss of strength during training.

In animal experiments the muscle behavior was also investigated with different frequencies. 20 Hz seem to have a positive influence on strength increases in the m. soleus of the mouse during a 14-day stimulation period (two times daily per 3 h of stimulation), furthermore positive influences on the development of the satellite cells can be determined (Guo et al., 2012). Exemplary studies showed an increased force production and tension with increasing frequency (Guo et al., 2012; Hering et al., 2016). However, an increased frequency cannot be maintained indefinitely. A constant stimulation with a higher frequency (e.g. 80 Hz or 100 Hz) results in a drop in force, which can be reduced by adjusting the frequency to 20 Hz for example. This fatigue during high frequency stimulation may be due to the failure of electrical propagation at the muscle fiber membrane, a reduction in the activity of the motor unit activity seems to minimize fatigue. A constant stimulation of 20 Hz generates a constant force diagram with a force increase after continuous stimulation. In comparison, in voluntary contraction the force generation is optimized by a reduction in motor neuron firing frequency to avoid this type of fatigue (Bigland-Ritchie et al., 1979; Jones et al., 1979).

On this basis, the aim of the present study was to observe the frequency-dependent response behavior of the triceps surae muscle of the mouse in EMS.

## Methods

**FIGURE 1:**
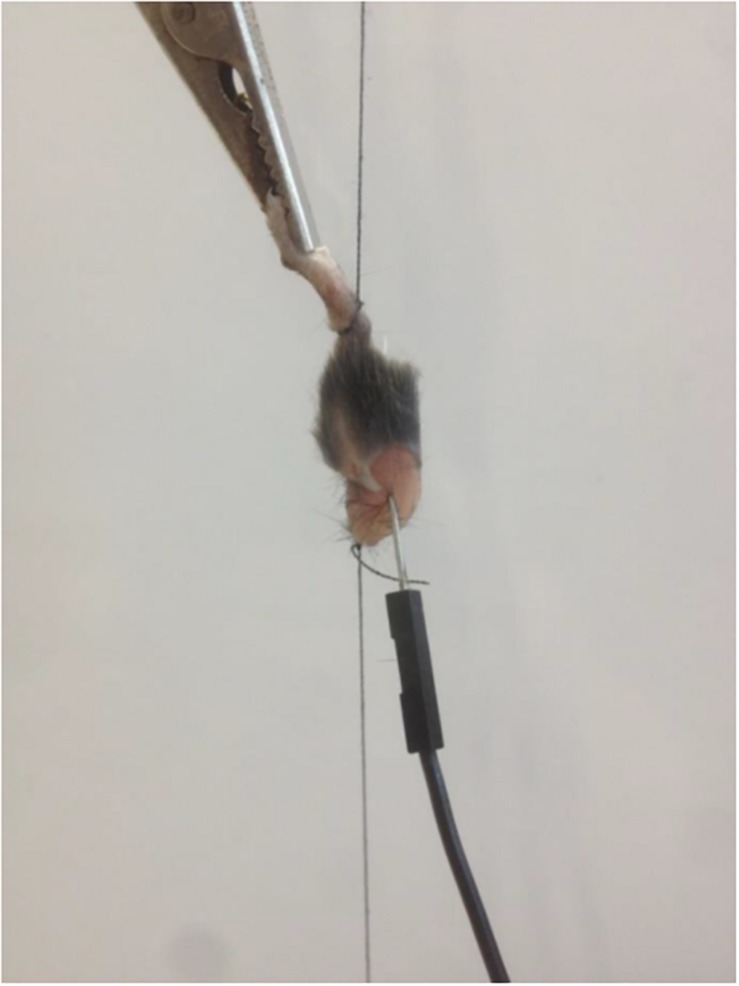
Fixed mouse muscle with needle and clamp electrode.

The experiments were performed on triceps surae muscle (m. soleus and m. gastrocnemius) of 19 wild type laboratory mice (C57BL/6N; age: 34.7 ± 2.2 days; weight: 17.2 ± 1.7 g). Animals were raised in the animal facilities of the University of Kaiserslautern under normal nutritional conditions. Lights were set to a 12 h day-12 h night cycle. Animal breeding and experiments were approved by the regional council according to the German animal protection act (TSchG §4, Absatz 3) and in accordance with EU Directive 2010/63/EU. To carry out the experiment, the neck of 19 mice was broken with the preparation scissors and then the head was severed. The lower leg was separated from the thigh 5 mm above the knee joint and the coat, skin and connective tissue were removed. The exact muscle fiber composition of the examined musculature is shown in Table 1, the myosin isoform percentage (MHC) in Table 2. The preparation was clamped vertically in the area of the tarsus as well as below the knee joint by two threads with the same pre-tension in a tripod apparatus (Figure 1).

**TABLE 1:** Percentage of muscle fiber types in the soleus and gastrocnemius muscle, modified according to Augusto et al. (2004).

Muscle fiber type	m. soleus [%]	m. gastrocnemius [%]
Type I	37.42	5.74
Type IIA	38.62	5.73
Type IID	5.69	2.26
Other type II (AD, DB and B)	18.74	86.19

**TABLE 2:** Myosin isoform percentages (MHC) in the soleus and gastrocnemius muscle, modified according to Augusto et al. (2004).

MHC	m. soleus [%]	m. gastrocnemius [%]
I	41.5	0.81
IIa	57.56	17.01
IId	0.15	0.00
IIb	0.00	84.50

After the preparation of the muscle, it was immediately clamped into the apparatus to avoid a long period of time between preparation and analysis. At the lower end a bending rod transducer with amplifier was fixed, the signal was amplified tenfold. The amplified signal was output and stored optically and digitally via an oscilloscope (Tektronix TDS1001B, Tektronix, Schwalbach am Taunus, Germany) at 250 Hz. For the stimulation a clamp electrode was fixed to the paw and a needle electrode was inserted through the muscle belly of the muscle. The sequence of the selected frequencies was randomized from muscle to muscle so each frequency was used at each measurement time point. The monopolar stimulation of the triceps surae muscle was performed by a self-developed stimulation generator which modulated the pulse duration (4 s), frequency (20, 60, 85, 100 Hz) and width (350 μs) for a rectangular signal. The resulting stimulation scheme largely corresponded to the electrical stimulation used in a whole-body-EMS (WB-EMS) application. In order to realize a stimulation at a desired current strength of 30 mA, a voltage of 8.7 V with a resistance of 270 Ohm was determined on the oscilloscope.

During the stimulations, the shortening of the triceps surae muscle was measured via the bending rod transducer as relative units (here designated by a.u. = arbitrary units). The present equilibrium length of the muscles before stimulation corresponded to the initial value of zero. Positive values during stimulation showed muscle shortening. Thus, a comparison between the frequencies was performed on the basis of the relative unit. We determined maximum and mean shortening and the integral over the time-length diagram in relation to frequency. The results are expressed as mean values and standard deviations.

We included only measurements of 15 mice or mouse muscles in the analysis because measurement errors occurred in four cases. The statistical analysis was performed with IBM SPSS (SPSS Version 25.0, Chicago, IL, United States). Because of missing normal distributions Kruskal-Wallis-Tests were performed to evaluate differences of mean value changes in relative muscle lengths between the frequencies. Follow up Mann-Whitney-U-tests were conducted to evaluate pairwise differences, controlling for Type I error across tests by using Bonferroni approach.

## Results

With regard to the frequency-dependent, averaged integrals, no significant differences in triceps surae muscle shortening could be observed depending on the selected frequency sequence. Therefore, we could exclude a sequence-related fatigue effect. In spite of different preloads due to randomized stimulation, we found the largest muscle shortenings at 20 Hz (Table 3).

**TABLE 3:** Averaged results across all mice for the frequencies 20, 60, 85, and 100 Hz.

Stimulation Frequency (Hz)	Integral (a.u.)	Mean (a.u.)	Maximum (a.u.)
20	13.27 ± 8.25	3.32 ± 2.06	3.71 ± 2.27
60	3.09 ± 2.32	0.77 ± 0.58	2.49 ± 1.64
85	1.27 ± 1.03	0.32 ± 0.29	1.68 ± 0.85
100	1.24 ± 1.14	0.31 ± 0.29	1.36 ± 1.00

*Values are reported as means ± standard deviations. a.u. = arbitrary unit.*

**FIGURE 2:**
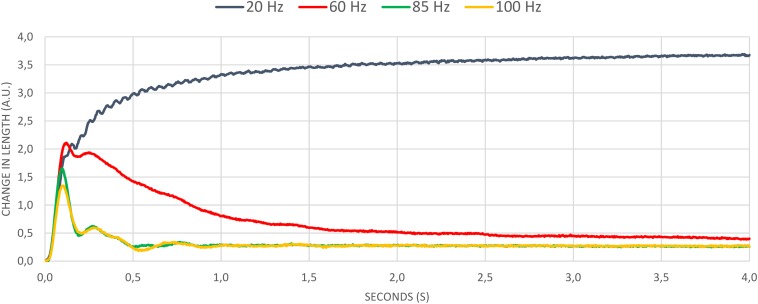
Mean values of all frequencies.

In addition, we found continuous muscle shortening (tetanus) during stimulation at 20 Hz. For frequencies above 50 Hz there was no permanent and constant muscle shortening, the muscle length increased again in the course of the stimulation. At 85 and 100 Hz this course was even more obvious in the graph (Figure 2). Already after 0.5 s, the muscle almost regained its equilibrium length.

The Kruskal-Wallis-Test revealed significant differences between the frequencies used (*p* < 0.050). The results of the Mann-Whitney-U-tests indicated a significant difference between 20 Hz and all other frequencies (all *p* < 0.050), but none in any other pairwise comparison.

## Discussion

In this study, the dissected triceps surae muscle of the mouse was subjected to muscle stimulation corresponding to WB-EMS training. Four frequencies were randomly applied and the respective stimulus response was determined by means of relative muscle length changes. On basis of this experimental setup with the same external weight load and pre-tension of the triceps surae muscle we found different muscle activation levels.

At 20 Hz, the largest change in muscle length was induced approximately over the full stimulation time. Only for this frequency a permanent and constant muscle shortening had occurred and thus a tetanic contraction could be assumed. The time-length-diagrams for the frequencies of 60, 85, and 100 Hz, on the other hand, showed less muscle shortening. The question now arises why we could not find an increased shortening at higher frequencies, what the force frequency relationship (FFR) suggests. Studies found an increase in muscle tension (Guo et al., 2012) or strength production (Hering et al., 2016) due to an increase in the frequency in animal experiments. First of all, it has to be taken into account that our experiments are carried out *ex vivo*, using a needle electrode, which complicates the comparability to *in vivo* or *in vitro* studies.

According to Augusto et al. (2004), 2–3 months old mice have a fiber composition with over 80% type II fibers in triceps surae muscle. Due to the young age of the mice used, the differentiation of the muscle fiber types are not complete, which could result in an insufficient number of motor units responding to high frequencies. It is known that muscle activity induced by EMS causes altered recruitment behavior in muscle fibers compared to voluntary contractions. Contrary to the activation sequence of Henneman’s size principle, studies with EMS show either a selective activation of fast motor units (Cabric et al., 1988; Trimble and Enoka, 1991) or a non-selective, spatially fixed and temporally synchronous recruitment pattern of muscle fibers (Bickel et al., 2011). In the first case, there might be no increase in muscle activity, since the muscle fibers of the growing mouse are not yet sufficiently differentiated and thus less fast-twitching fibers can be activated. In addition, one could assume that most muscle fibers had already reached their stimulation threshold at low frequencies and therefore no increase in muscle activity and further shortening of muscle length could be achieved at higher frequencies. In the second case, slow and fast muscle fibers are activated at both low and high strength levels (Gregory and Bickel, 2005). Thus, according to Bickel et al. (2011), stimulation with low (20 Hz) and high frequency (>50 Hz) should have activated all types of muscle fibers.

However, it is assumed that higher frequencies may lead to faster muscle fatigue, as during EMS it is not possible to reduce the innervation frequency or modulate the recruitment pattern through physiological control processes (Gregory and Bickel, 2005; Dreibati et al., 2010). Experimental investigations on the soleus muscle of the mouse show exactly this behavior by means of the force progression. At 100 Hz stimulation frequency, the force drops to 10% of the initial value after 40 s. In contrast, force remains at the same level for at least 60 s when stimulated at 20 Hz (Jones et al., 1979). Compared to the results presented in this study, only the time of fatigue differs significantly. The much earlier decrease in muscle activity could be explained by the experimental setup. In the study by Jones et al. (1979), the mouse muscle is placed in a sodium solution so that an ion exchange between muscle tissue and fluid is possible. In the present study, the muscle was isolated and separated from any circulation, which would allow the fatigue processes to progress more rapidly. Nevertheless, the graphs of force development at different frequencies from Bigland-Ritchie et al. (1979) show very strong similarities to the *ex vivo* results presented here. A continuous force development at 20 Hz can be seen, whereas higher frequencies (50 Hz and 80 Hz) already after a short stimulation time of about 5 s lead to a continuous decrease in force production, which remains constant until the end of the stimulation (Bigland-Ritchie et al., 1979). Furthermore, frequency modulation from 100 Hz to 20 Hz after continuous stimulation can in turn bring about a clear increase in force (Jones et al., 1979).

With regard to the behavior of human muscles in comparison to mouse muscles during EMS, studies show the same fatigue behavior (Jones et al., 1979; Moritani et al., 1985). As a reason for the decrease in strength during stimulation with high frequencies, it is assumed that the transmission of the action potentials via the T-tubules is no longer possible and therefore the strength production collapse. A changed ion concentration leads to a reduction of the membrane excitability and thus to a lower power development (Bigland-Ritchie et al., 1979). Binder-Macleod and McDermond (1992) show that the contraction rate of skeletal muscles slows down when tired muscles are excited voluntarily or by EMS. This means that although muscle twitching is faster when stimulated electrically, the half relaxation time is significantly longer. Thus, at high frequency, a new electrical stimulus occurs during the repolarization phase and therefore remains ineffective. This could signify for WB-EMS that lower frequencies guarantee an adequate repolarization time of the muscle fiber membrane especially in a fatigued state, which could be important for the periodization of the WB-EMS training in competitive sports. At a higher frequency, an increased metabolic demand leads to a faster fatigue of the muscles. However, in WB-EMS this could be an important factor for modulating the applied frequency during the training from a higher to a lower frequency (Dreibati et al., 2010).

The study carried out here showed the highest force development at a frequency of 20 Hz. This could be a further indication of an increased metabolic need of the muscle at higher frequencies, since the musculature could only cause a continuous contraction at 20 Hz. Only at higher frequencies a lower force impulse could be generated. A decisive factor here, however, is the conduct of the study in an *ex vivo* experimental design. Nevertheless, other authors such as Glaviano and Saliba (2016) or Dreibati et al. (2010) also describe an improved force production at lower frequencies and stated an increased metabolic demand in higher frequencies (ph level, inorganic phosphocreatine values, energy costs), which would confirm our results due to the lack of new energy production in the *ex vivo* muscle. A further reason for the strength loss with increased frequencies could be the reduction in the extracellular Na^+^ due to the shorter action time for the sodium-potassium pump. The depletion of the Na^+^ (or the accumulation of K^+^) could reduce the muscle membrane excitability sufficiently to explain the force loss during a higher frequency (Moritani et al., 1985).

Due to different stimulation protocols and other environmental conditions, the comparison of the results presented here to other animal studies remains complicated (Guo et al., 2012; Kobayashi et al., 2012; Tsutaki et al., 2013; Hering et al., 2016; Li et al., 2016; Valenzuela et al., 2017). Nevertheless, similar results could be observed in the behavior of the muscle during stimulation with different frequencies, even if the environmental influences and the way of stimulation of the muscle (*ex vivo*, needle electrode) were different. In future experiments, the influence of surface electrodes and needle electrodes on stimulation behavior of the muscle should be investigated. Since comparative studies in animals use surface electrodes in the experiments, this could be a source of interference in the experimental setup. It should also be examined to what extent an examination with intact blood circulation would generate similar muscle behavior. In addition, the relationship between stimulation frequency and intensity should be further examined in a combined study protocol. Nevertheless, the available results provide an insight into the behavior of the *ex vivo* mice muscles at different applied frequencies.

In addition to the limitations described such as the use of a needle electrode instead of surface electrodes and the *ex vivo* implementation of the study without nutrient supply for the muscle, the lack of knowledge about the MHC isoform of the muscles used is a further limitation. Although comparisons can be made with the studies carried out by Augusto et al. (2004), future studies should carry out a detailed MHC isoform analysis of the examined muscles in each case in order to be able to interpret possible fluctuations of the muscle fiber composition (Zhang et al., 2010) and corresponding divergent reactions to an electrical stimulus more accurately.

The frequency used seems to have a significant influence on fatigue, which must also be considered in the context of training planning for WB-EMS. Since active movements of the athlete (e.g. light strengthening exercises) lead to greater muscle strength compared to passive WB-EMS, the frequency should be chosen accordingly in order to be able to perform the movements in a controlled manner throughout the training (Kemmler et al., 2018).

## Data Availability Statement

The raw data supporting the conclusions of this article will be made available by the authors, without undue reservation, to any qualified researcher.

## Ethics Statement

This study was carried out in accordance with the recommendations of the regional council according to the German animal protection act (TSchG §4, Absatz 3) and in accordance with EU Directive 2010/63/EU. The protocol was approved by the regional council.

## Author Contributions

SZ, JB, JK, and MF conceived and designed the experiments. JK performed the experiments. JK, SZ, and JB analyzed the data. SZ and MF contributed materials and analysis tools. SZ, JB, and OL wrote the manuscript.

## Conflict of Interest

The authors declare that the research was conducted in the absence of any commercial or financial relationships that could be construed as a potential conflict of interest.
